# Prevalence and correlates of modifiable risk factors for cervical cancer and HPV infection among senior high school students in Ghana: a latent class analysis

**DOI:** 10.1186/s12889-022-14908-w

**Published:** 2023-02-15

**Authors:** Ama Gyamfua Ampofo, Allison W. Boyes, Shadrack Osei Asibey, Christopher Oldmeadow, Lisa J. Mackenzie

**Affiliations:** 1 Health Behaviour Research Collaborative, School of Medicine and Public Health, College of Health, Medicine and Wellbeing, The University of Newcastle, Callaghan, NSW 2308 Australia; 2 Equity in Health and Wellbeing Research Program, Hunter Medical Research Institute, New Lambton Heights, NSW Australia; 3 Hunter Medical Research Institute, New Lambton Heights, NSW Australia; 4 Faculty of Applied Sciences and Technology, Kumasi Technical University, Kumasi, Ghana

**Keywords:** Uterine cervical neoplasms, Risk factors, HPV infection, Latent class analysis, Ghana, Schools, Adolescents

## Abstract

**Background:**

While health risk behaviours are likely to co-occur, there is dearth of studies exploring the clustering of cervical cancer and HPV infection risk factors among adolescents. This study aimed to determine: 1) the prevalence of modifiable risk factors for cervical cancer and HPV infection, 2) the clustering of modifiable risk factors for cervical cancer and HPV infection, and 3) factors associated with the identified clusters.

**Methods:**

Female students (aged 16–24 years, *N* = 2400) recruited from 17 randomly selected senior high schools in the Ashanti Region, Ghana completed a questionnaire assessing modifiable risk factors for cervical cancer and HPV infection including sexual experience, early sexual intercourse (< 18 years), unprotected sex, smoking, sexually transmitted infections (STIs); multiple sexual partners (MSP) and smoking. Latent class analysis explored separate classes of students according to their risk factor profiles for cervical cancer and HPV infection. Latent class regression analysis explored factors associated with latent class memberships.

**Results:**

Approximately one in three students (34%, 95%CI: 32%-36%) reported exposure to at least one risk factor. Two separate classes emerged: high-risk and low-risk (cervical cancer: 24% and 76% of students, respectively; HPV infection: 26% and 74% of students, respectively). Compared to participants in the low-risk classes i) the cervical cancer high-risk class were more likely to report exposure to oral contraceptives; early sexual intercourse (< 18 years); STIs; MSP and smoking; and ii) the HPV infection high risk class were more likely to report exposure to sexual intercourse; unprotected sex and MSP. Participants with higher risk factor knowledge had significantly higher odds of belonging to cervical cancer and HPV infection high-risk classes. Participants with greater perceived susceptibility to cervical cancer and HPV infection were more likely to belong to the high-risk HPV infection class. Sociodemographic characteristics and greater perceived seriousness about cervical cancer and HPV infection had significantly lower odds of belonging to both high-risk classes.

**Conclusions:**

The co-occurrence of cervical cancer and HPV infection risk factors suggests that a single school-based multi-component risk reduction intervention could concurrently target multiple risk behaviours. However, students in the high risk class may benefit from more complex risk reduction interventions.

**Supplementary Information:**

The online version contains supplementary material available at 10.1186/s12889-022-14908-w.

## Background

Cervical cancer incidence and mortality are increasing in sub-Saharan African countries such as Ghana. Approximately 70% of these cases are caused by potentially modifiable sexual and non-sexual behavioural risk factors [1]. Sexual behaviour factors include early age at first sexual intercourse (< 18 years), multiple sexual partners, sexually transmitted infections (i.e., Human immunodeficiency virus (HIV) and Human Papillomavirus (HPV), and non-sexual behavioural factors include tobacco smoking and long-term oral contraceptive usage (more than five years) [1]. Sexual behavioural factors are likely to increase susceptibility to and acquisition of HPV infections [2]. In Ghana, approximately half of all cervical cancer cases are attributed to HPV types 16/18 [1, 3, 4]. The highest prevalence (41.7%) of high risk HPV infection (including HPV type 16) is among women younger than 25 years [3].

Given that health risk behaviours often co-occur or tend to cluster together within populations, their synergistic effect may increase the risk of disease or mortality compared to single risk factors [5]. Exploring clusters of risk behaviours through techniques such as latent class analysis allows groups of individuals exhibiting similar behaviours, the size of the groups and the characteristics associated with the groups to be identified. There is a growing body of evidence of the clustering of health behavioural risk factors for various health conditions across countries, and the characteristics of identified groups, among adolescents. Cancer risk behaviours (tobacco use, physical inactivity, unhealthy diet, alcohol binge drinking, and overweight/obesity) were reported to cluster among college students in America by race and ethnicity [6]. In Brazil, physical inactivity, excessive alcohol consumption, smoking, sedentary behaviour and unhealthy diet clustered for non-communicable diseases; and girls, older adolescents and people from low socioeconomic status were more likely to belong to high risk groups [7].

Adolescence and young adulthood are critical periods in the life course for developing health behaviour patterns that have implications later in life [8]. More than 40% of school-age adolescents in Ghana are sexually experienced [9–11] and median age of first sexual intercourse among high school students is 15 years [12, 13]. In Ghana, females are five times more likely than males to initiate sex early [13]. While studies have provided separate prevalence estimates of other risky behaviours including having unprotected sex (> 60%) [9, 11], multiple sexual partners (> 30%) [10, 11], and smoking (> 6%) [3, 12, 14] alone, the evidence for school girls is limited.

According to the Health Belief Model, knowledge, beliefs and perceptions about a disease are likely to influence health behaviours including risky behaviours about cervical cancer and HPV infection [15]. Previous studies have reported conflicting association between knowlegde and perceptions, and risky behaviours for cervical cancer and HPV infection among adolescent girls and young women. While sexually active adolescents and young women in South African communities and tertiary institutions perceived themselves at high risk of cervical cancer [16], female students with increased sexual risk behaviours perceived themselves a low level of risk in America [17, 18]. Denny and collegues also reported that female students in America who perceived cervical cancer and HPV infection as less severe were engaged in high-risk behaviours [17]. In Nigeria, women with multiple sexual partners are less likely to have good knowledge about cervical cancer [19]. Although these studies provide evidence to support the knowledge-perception-behaviour gap, they had methodological shortcomings including small sample sizes (between 122 and 200) and use of convience sampling techniques [16–18]. They also assessed a limited range of cervical cancer and HPV infection risk factors.

To the authors’ knowledge, no study has examined the clustering of comprehensive cervical cancer and HPV infection risk factors, and associated demographics, perceptions and knowledge of the disease among adolescents in Ghana. Understanding whether, how and which risk factors cluster together has several applications in health promotion. These include: i) identifying subgroups within the population with a higher risk of disease, greater risk for future disease conditions or death due to engaging in multiple health risk behaviours; ii) targeting different groups according to their specific characteristics, and relationships within contexts; iii) tailoring, designing and implementing health promotion strategies to particular settings and iv) avoiding overlaps between various health promotion programmes targeted at disease prevention implemented in the same context [20, 21]. Furthermore, understanding perceptions and knowledge about cervical cancer and HPV infection are important for behavioural changes leading to engagement in health-promoting activities such as reducing risk exposure, participating in screening and vaccination [15, 22].

Given the gap in evidence, we examined among female senior high school students in Ghana a) the prevalence of a range of modifiable risk factors for cervical cancer and HPV infection; and b) the clustering of modifiable risk factors for i) cervical cancer and ii) HPV infection; and c) explored the factors (i.e., participant characteristics, perceived susceptibility to and seriousness of, and knowledge about cervical cancer and HPV infection) associated with the identified risk clusters.

## Methods

### Study design, setting and period

This cross-sectional study was conducted in public senior high schools recognised by the Ghana Education Service (GES) in the Ashanti Region between February-July 2021. The Ashanti region has the second-highest population of Ghanaians and the highest number of senior high schools in Ghana [23]. Public schools admit the highest number of students in Ghana [23].

### Participants

#### Criteria for inclusion of schools and students

Schools were recruited if they were mixed and single-sex (all girls) public senior high schools (SHS). Students were eligible if they were: female, 16 years and older, and could provide parent/guardian consent.

#### Sampling

A two-stage sampling process was performed. Cluster sampling was used to randomly select eligible schools from 122 public institutions for the first stage. In the second stage, quota sampling was used to recruit eligible students from selected schools.

#### Sample size

A sample of 17 schools with an average of 71 eligible student participants per school would enable estimation of the proportion of students with a risk factor (assumed to be 50%) with a margin of error of 6%. This calculation assumes an effective sample size of 260 students, resulting from a design effect of 4.55 and an intra-class correlation of 0.05. Allowing for a conservative 50% response rate, an average of 141 consenting students per school was estimated to give a final sample size of 2400 participants.

### Procedure

#### Recruitment of schools

Prior to the recruitment of schools, an arbitrary horizontal line was used to divide the region into northern and southern sectors to ensure adequate representation of students in the region (see Supplementary (S) Figure S1). Schools were further stratified by location [(District (95,000 > x > 75,000 inhabitants) or Municipal (250,000 > x > 95,000 inhabitants)/Metropolitan (> 250,000 inhabitants)]. This process resulted in 4 strata: north District, north Municipal/Metropolitan, south District, and south Municipal/Metropolitan schools.

An independent statistician automatically generated and assigned a random number using a Microsoft Excel spreadsheet to each school in the strata. The generated random number was sorted from smallest to biggest, and the first four schools from each stratum were selected (*n* = 16 schools). For the seventeenth school to be chosen, one of the four strata was chosen randomly, and a fifth school was selected from the stratum. The seventeen included schools consisted of two single-sex girls’ schools and fifteen co-education schools.

Permission to approach schools about the study was sought from the District Directorates of the eligible schools. Information about the study was provided to the heads of the school, and written consent for the school to participate in the study was obtained. The first four schools from the randomly generated list in each stratum were approached and invited to participate by members of the research team. If a school declined to participate in the study, the next school was approached and invited until the total number of consenting schools (*n* = 17) was achieved.

#### Recruitment of students

Trained local research assistants verbally informed students at consenting schools about the study during the schools' general assembly. Eligible students were invited to visit the school’s study-designated classroom during their break periods. Following Public Health orders in response to the COVID-19 pandemic, tables and chairs in the designated classroom were arranged 1.5 m apart and masks, sanitiser and pens were provided to students to maintain their health and safety. Students who came to the designated classroom were invited to complete an anonymous pen-and-paper survey assessing sociodemographic characteristics, behavioural risk factors for cervical cancer and HPV infection, knowledge and perceptions about cervical cancer (see Measures).

Verbal and written statements were made clear to students that by completing the survey they were providing consent to participate in the study. Students were also made aware that their decision to participate was completely voluntary and that their responses were anonymous; they were allowed to withdraw from the survey at any time and skip any question they did not wish to answer. To ensure students' confidentiality and privacy, they were asked to put completed surveys in a sealed envelope and place them in allocated boxes at the front of the room. Neither heads of schools nor teachers knew which students completed the survey. For students under 18 years, written parental/guardian consent was obtained before surveys were conducted. Recruitment continued in all 17 schools until the targeted number of at least 141 students per school was achieved.

### Measures

A study-specific survey was developed based on a review of qualitative and quantitative studies about cervical cancer knowledge beliefs and attitudes, and a youth risk behaviour surveillance system (YRBSS) questionnaire [22, 24–27]. Input was obtained from health behaviour scientists (*n* = 4), epidemiologists (*n* = 2), and healthcare providers including nurses (*n* = 2), doctors (*n* = 2) and psychologists (*n* = 2) to ensure face and content validity. The survey was piloted in two phases among 100 students in a public senior high school in the Ashanti Region. The first phase tested all the survey steps including administration, data entry and analysis. In the second phase, 20 students were purposively sampled, and semi-structured telephone interviews were conducted by the lead author (AGA) to seek feedback about the survey and explore their perceptions and beliefs about cervical cancer and HPV infection.

Based on the results of the pilot study and further review by behavioural scientists and healthcare experts, changes were made to the survey including adding of new items, deletion of redundant or duplicate items, simplifying complex items, re-ordering items, and revising response options. The psychometric performance of the scale was evaluated [28]. Inter-item, and item-total correlations indicated that item efficiency was good, with a good correlation among all items (*r* = 0.2 to 0.7) [29]. Item-by-item frequency distribution showed a good spread, and internal consistency reliability values were acceptable (Cronbach’s alpha: 0.60 to 0.82) [29]. The final outcome measure comprised the following components (see S2 for full details):

#### Outcomes

*Cervical cancer behavioural risk factors* were assessed using a single standardised item for each risk factor from the YRBSS questionnaire [24], which demonstrated good reliability [30] including *i) Age of first sexual debut;* response options were “16 years or younger/17 years or older/Prefer not to answer” *ii) Number of sexual partners;* response options included “I have never had sexual intercourse before/one/ two or more/ prefer not to answer” *iii) Sexually transmitted infections; r*esponse options were “Yes/No/prefer not to answer” iv*) Oral contraceptives use*; response options were “I have never had sexual intercourse before/Yes/No/Prefer not to answer” *v) Smoking;* response options were “Yes/No”.

*HPV infection risk factors* were assessed using a single standardised item from the YRBSS questionnaire [24] including *i) Sexual intercourse;* response options were “Yes/No/Prefer not to answer” *ii) Unprotected sexual intercourse;* response options were “I have never had sexual intercourse before/Yes/No/Prefer not to answer” *iii) Multiple sexual partners*: as described above.

#### Covariates

The following factors which have been consistently identified in the literature as determinants for behaviour were assessed. *Participants’ characteristics:* One item assessed age; ethnicity (Akan/Northerner/Ewe and Guans/Ga-Adangbe/other); religion (Christian/Islam/other); relationship status (single/dating/other); living arrangements (both parents/one parent/grandparent(s)/guardian/ Friend(s)/on my own); Mother/female guardian and father/male guardian employment status (employed/unemployed).

*Cervical cancer and HPV infection risk factor knowledge* were assessed using 12 items. Participants were asked to indicate whether a particular item was associated with cervical cancer or not. There were six correct statements (e.g., smoking cigarettes, having sex before 18 years of age) and six incorrect statements (e.g., spiritual forces, having one or more abortions). Response options were “Yes/No” or “True/False”. The Cronbach alpha was 0.76, indicating adequate internal consistency. The number of correct responses was calculated, resulting in a score ranging from 0 to 12. A higher score represented a higher level of knowledge.

*Knowledge about primary prevention of cervical cancer and HPV infection was* assessed using five items. Participants were asked to indicate whether a particular item was a primary prevention strategy. There were three correct statements (e.g., getting vaccinated with HPV vaccines) and two incorrect statements (e.g., praying to God). Response options were True/False. The Cronbach alpha was 0.60, indicating acceptable internal consistency. The number of correct responses was calculated, resulting in a score ranging from 0 to 5. A higher score represented a higher level of knowledge.

*Perceived susceptibility to cervical cancer and HPV infection* was assessed using six items (four on cervical cancer and two on HPV infection). Participants were asked to rate their likelihood of getting cervical cancer and HPV infection on a 5-point Likert scale (1-Very unlikely to 5-Very likely). For example, “*How likely are you to develop cervical cancer?”.* The Cronbach alpha was 0.60, indicating acceptable internal consistency. Weighted composite scores were estimated using the partial credit model [31] with higher scores indicating higher perceptions of susceptibility.

*Perceived seriousness of cervical cancer and HPV infection* was assessed using eleven items (four items on HPV infection and seven items on cervical cancer). Participants were asked to rate their feelings about cervical cancer and HPV infection and whether they could cause death and affect their closest relationship and life span on a 5-point Likert scale (1-Strongly disagree to 5-Strongly agree). For example, “*Getting cervical cancer would reduce the number of years I live”.* The Cronbach alpha was 0.82, indicating a good internal consistency. Weighted composite scores were estimated using the partial credit model [31] with higher scores indicating higher perceptions of seriousness.

### Data analysis

Data were analysed using Stata statistical software version 16.1 (StataCorp, College Station, Tx). Participant characteristics and prevalence of risk factors were summarized as frequencies and percentages. Perceptions and knowledge were described as means and standard deviations. Due to small cell counts and the interpretability of findings, the response categories of some covariates were combined, including relationship status (single/dating and other); living arrangements (at least a parent/with no parent). Level of significance and confidence intervals (CI) were set at 5% and 95%, respectively.

The prevalence of cervical cancer risk factors was calculated for each participant, including having: used oral contraceptives to prevent pregnancy; had sexual intercourse before 18 years; had multiple sexual partners at any given time; tested positive for STI before and smoked before. The prevalence for HPV infection risk factors was also calculated for each participant, including having had sexual intercourse before, multiple sexual partners and unprotected sex.

Latent class analysis (LCA) was used to identify classes of risk factors for a) cervical cancer and b) HPV infection. LCA is a statistical tool used to identify homogeneous, mutually exclusive classes (or clusters) within a heterogeneous population using categorical variables [32]. Latent class models were fitted starting with four classes and then reducing the number of classes for each subsequent model. The goodness of fit and interpretability of the clusters were used to decide the optimal number of classes. The optimal number of classes was determined using the principle of parsimony and information criteria, including the Bayesian and Akaike Information Criterion (BIC and AIC). The LCA model was fit over a range of class numbers; BIC and AIC were generated for each (with lower BIC and AIC suggesting better goodness of fit) [33]. Descriptive statistics (conditional probabilities and proportions) were computed to explore the patterns of risk factors in each class membership [34]. The classes were given a descriptive name based on the proportions and probabilities of risk factors.

Latent class regression analysis was used to examine the association between covariates (i.e., participant characteristics, perceived susceptibility to and seriousness of and knowledge about cervical cancer and HPV infection) and identified class membership. This model permits the inclusion of covariates to predict individuals’ latent class membership [35]. Pearson correlation was used to test for multicollinearity for all continuous variables.

## Results

### Participants’ characteristics

All 17 schools approached agreed to participate (100% consent rate). As shown in Table 1, the mean age of the student participants was 17, and the majority were boarders (76%). Over 70% of participants were Akans, and 60% lived with their parents. More than 90% were Christians, and 70% were single.

**Table 1:** Participants’ characteristics, *N* = 2,400

Variable	N^a^ (%)
*Age, Mean(SD),* 17.32 (1.22)	
16 years	710 (30%)
17 years	771(32%)
18 years	559 (23%)
*19* + *years*	360 (15%)
*Ethnicity*	
Akan	1,860 (77%)
Northerners	300 (13%)
Ewe/Guans	121 (5%)
Ga-Adangbe/other	115 (5%)
*Enrolment status*	
Boarders	1,821(76%)
Non-boarders	560 (24%)
*Religion*	
Christianity	2,230 (93%)
Islam	153 (6%)
Other	13 (1%)
*Relationship status*	
Single	1,689 (71%)
Dating	648 (27%)
Other	56 (2%)
*Employment status of mother/female guardian*	
Employed	1,833 (77%)
Unemployed	544 (23%)
*Employment status of father/male guardian*	
Employed	1,910 (80%)
Unemployed	485(20%)
*Living arrangement*	
Both parents	1,442 (60%)
One parent	560 (23%)
Grandparent (s)	174 (7%)
Guardian	124 (5%)
Friend(s)	44 (2%)
On my own	51 (3%)

^a^May not add to 2,400 due to missing data

### Knowledge and perception of cervical cancer and HPV infection

As shown in Table 2, the mean risk factor and primary prevention knowledge scores were 5.73 (SD = 1.42) and 3.29 (SD = 1.41), respectively. The unweighted mean perceived susceptibility to and seriousness of cervical cancer and HPV infection score were 16.84 (SD = 4.09) and 39.07 (SD = 7.73) respectively. Perceived susceptibility included participants’ perceptions about the likelihood of developing cervical cancer and HPV infection in their life, whether their sexual activities and family background could lead them to get cervical cancer and HPV infection. Perceived seriousness included beliefs about whether cervical cancer and HPV infection would affect their relationships, school and cause death.

**Table 2:** Knowledge and perception of cervical cancer and HPV infection

*Variable*	Mean (SD)	Min	Max
Cervical cancer and HPV infection risk factor knowledge score	5.73 (1.42)	1	11
Primary prevention of cervical cancer and HPV knowledge score	3.29 (1.41)	0	5
Perceived susceptibility to cervical cancer and HPV infection score (6 items)	0.00 (0.78) ^a^ 16.84 (4.09)^b^	-2.16 6	2.58 30
Perceived seriousness of cervical cancer and HPV infection score (11 items)	0.00 (0.91) ^a^ 39.07 (7.73)^b^	-3.13 11	2.62 55

^a^ Weighted composite score: higher scores represent greater perceptions and beliefs

^b^ Unweighted composite score: higher scores represent greater perceptions and beliefs

### Prevalence of self-reported modifiable risk factors for cervical cancer and HPV infection

As seen in Table 3*,* the most prevalent risk factors for cervical cancer were sexual intercourse before 18 years (16%, 95%CI: 14%-17%) and the use of oral contraceptives (15%, 95%CI: 14%-17%). For HPV infection, 28% (95%CI: 27%-30%) of participants reported having had sexual intercourse before. However, among those who reported having had sexual intercourse, 407(60%) did not use a condom, about half (*n* = 313, 46%) had sex before the age of 18, and more than 20% had: Multiple sexual partners (*n* = 156, 23%); had been diagnosed with an STI (*n* = 21%) (not shown in table). More than 30% of participants had been exposed to at least one risk factor for cervical cancer and HPV infection.

**Table 3:** Prevalence of self-reported modifiable risk factors for cervical cancer and HPV infection

Variable	N (%)	95%CI
*Cervical cancer risk factor*		
Sexual intercourse before 18 years	379 (16%)	(14%-17%)
Use of oral contraceptives to prevent pregnancy	364 (15%)	(14%-17%)
Sexually transmitted infection	269 (11%)	(9%-13%)
Multiple sexual partners (two or more)	186 (8%)	(7%-8%)
Smoking	176 (7%)	(6%-8%)
*Risk for HPV infection*		
Sexual intercourse	682 (28%)	(27%-30%)
Multiple sexual partners (two or more)	186 (8%)	(7%-8%)
Unprotected sexual intercourse	532 (22%)	(21%-24%)
*Exposure to co-occurring cervical cancer risk factors*		
No risk factors	1,587 (66%)	(64%-68%)
One or more risk factors	813 (34%)	(32%-36%)
*Exposure to co-occurring HPV infection risk factors*		
No risk factors	1,579 (66%)	(64%-68%)
One or more risk factors	821 (34%)	(32%-36%)

### Classes and patterns of risk factors for cervical cancer and HPV infection

**Fig. 1:**
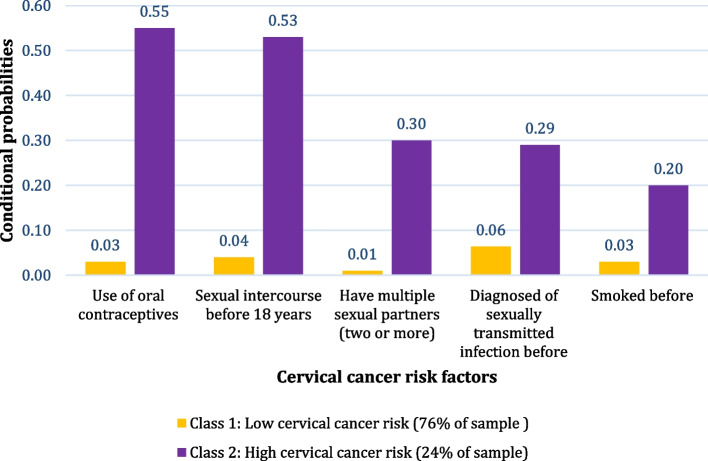
Latent profiles of cervical cancer risk factors of class membership

**Fig. 2:**
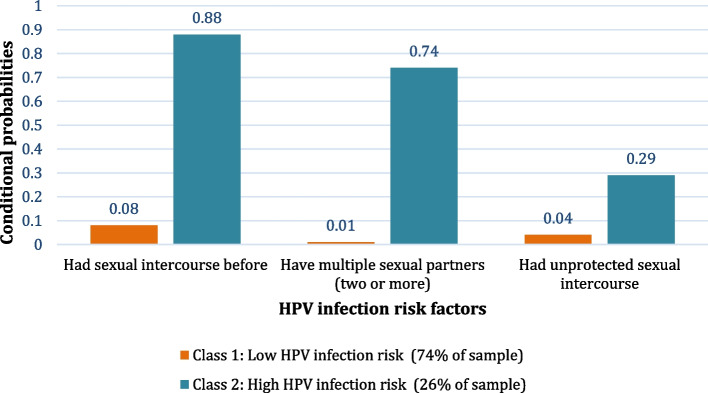
Latent profiles of HPV infection risk of class membership

Latent classes for models one to four were fitted without covariates for cervical cancer risk factors and one to three latent classes for HPV infection risk factors. The 2-class model was considered the best fit for the data on cervical cancer because it had the lowest BIC (7807.983) and AIC (7744.367). For HPV infection, the 2-class model was also considered the best fit, with a lower BIC (5707.226) and AIC (5666.744). The conditional probabilities of each risk factor associated with the classes are summaries below and shown in Figs. 1 and 2:

#### Two classes for cervical cancer risk factors were characterised as low and high risk

The low cervical cancer risk group comprised of participants (76%) with the lowest probability of using oral contraceptives to prevent pregnancy, sexual intercourse before 18 years, testing positive for STI, having multiple sexual partners (two or more) and smoking. Participants in the high cervical cancer risk group (24%) had the highest probability of all risk factors mentioned above (see Fig. 1).

#### Two classes for HPV infection risk factors were characterised as low and high risk

The low HPV infection risk group (74%) consisted of participants with the lowest probability of having had; sexual intercourse, unprotected sexual intercourse, and multiple sexual partners (two or more). Participants in high HPV infection risk group (26%) had the highest probability of all risk factors mentioned above (see Fig. 2).

### Correlates of cervical cancer risk class

Compared to the low cervical cancer risk group, participants with higher risk factor knowledge scores had significantly higher odds of belonging to the high cervical cancer risk class. On the other hand, those who were single, a boarder, and had higher perceived seriousness of cervical cancer and HPV infection scores had significantly lower odds of belonging to the high cervical cancer risk class *(*see Table 4).

**Table 4:** Knowledge and perceptions about cervical cancer and HPV infection: Sociodemographic characteristics associated with membership of “high cervical cancer risk” and “high HPV infection risk” latent classes

Variable	AOR, 95%CI	
	Class 2: High cervical cancer risk	Class 2: High HPV infection risk
*Enrolment status*		
Boarders	0.71 (0.51–0.98)^a^	0.92 (0.69–1.24)
Non-boarders	Reference	Reference
*Relationship status*		
Single	0.08 (0.06–0.11)^a^	0.09 (0.07–0.12)^a^
Dating/other	Reference	Reference
*Employment status of mother/female guardian*		
Employed	0.82 (0.54–1.23)	0.61 (0.42 -0.89)^a^
Unemployed	Reference	Reference
*Employment status of father/male guardian*		
Employed	0.71 (0.46–1.10)	0.96 (0.65–1.41)
Unemployed	Reference	Reference
*Living arrangements*		
Living situation: with at least one parent	0.72 (0.50–1.03)	0.58 (0.42–0.79)^a^
Living situation: with no parent	Reference	Reference
Perceived susceptibility to cervical cancer and HPV score	1.20 (0.99–1.45)	1.32 (1.12–1.56)^a^
Perceived seriousness of cervical cancer and HPV score	0.72 (0.61–0.85)^a^	0.83 (0.72–0.97)^a^
Cervical cancer and HPV risk factor knowledge score	1.21 (1.09–1.33)^a^	1.09 (1.00–1.19)^a^
Primary prevention of cervical cancer and HPV knowledge score	1.02 (0.93–1.14)	1.02 (0.94–1.12)

^a^Significant, *AOR* Adjusted Odds ratio, *CI* Confidence interval, Reference classes: classes 1- Low cervical cancer and HPV infection risk classes

### Correlates of HPV infection risk class

Compared to the low HPV infection risk class, participants with higher perceived susceptibility to cervical cancer and HPV infection scores and higher risk factor knowledge scores had significantly higher odds of belonging to the high HPV infection risk cluster. Conversely, those who were single, had their mother/female guardian employed, lived with at least one parent, and had a higher perceived seriousness of cervical cancer and HPV infection had significantly lower odds of belonging to the high HPV risk class (see Table 4).

## Discussion

Adolescence and young adulthood are critical periods for developing healthy behaviours. However, high school students engage in a range of risky behaviours that can lead to cervical cancer despite the burden of the disease. This study found a high prevalence of cervical cancer risk factors (oral contraceptives use and sexual intercourse before 18 years) and HPV infection risk factors (sexual intercourse and unprotected sex) among female Ghanaian high school students. Two in five female students reported being exposed to at least one risk factor. This finding is consistent with previous studies among girls in the Greater Accra and Volta regions of Ghana [9, 10, 12, 36] and supported by studies reporting these risk factors among both sexes in the Central and Bono-East regions of Ghana [10, 11, 13]. The emergence of two high-risk classes for cervical cancer and HPV infection with more than 20% of participants confirmed this finding. Within these classes, nearly all the risk factors assessed were highly prevalent. The co-occurrence of cervical cancer and HPV risk factors suggest that single multi-component risk reduction interventions could concurrently target multiple risk behaviours. The significant distinction between high and low risk classes indicates that students who are at increased risk are likely to benefit from complex risk reduction interventions. These strategies may include risk education, risk sensitization and behaviour self-management and communication skills building [37] (including safer sex practices—buying, carrying and use of condom, condom negotiation; delaying sexual activities; and smoking cessation/reduction).

To the best of our knowledge, no previous work has explored clusters of cervical cancer and HPV infection risk factors in female high school students. However, our findings were compared to studies reporting health risk behaviours in general. In line with our study, a 1970 British cohort study identified two latent classes (high and low risk) for health risk behaviours (smoking, drinking, drug use, early sexual intercourse, unprotected sex, physical inactivity, involvement in fights and delinquency) among 16-year-old adolescents girls [38]. Those in the high-risk group exhibited a high probability of smoking, sexual debut before 16 years, and unprotected sex [38]. This suggests that cervical cancer education should be implemented for early adolescents (at the primary and junior high school levels), including behavioural risk reduction strategies and teaching methods for condom use and smoke cessation.

We found a knowledge-perception-behaviour gap among students as those with higher risk factor knowledge are more likely to belong to high-risk cervical cancer and HPV infection clusters. Also, students who perceived themselves as susceptible to cervical cancer and HPV infection were more likely to engage in HPV infection risk behaviours. Similar to past cross-sectional studies, female college students with greater perceived susceptibility of [17, 18] and high knowledge about cervical cancer/HPV infection [17] were more likely to have multiple sexual partners in the past year than those with lesser perceived susceptibility and lower knowledge. The knowledge-perception-behaviour gaps suggest the presence of barriers to changing behaviour that can prevent cervical cancer and HPV infection among Ghanaian senior high students.

Although some students can recognise their risks, aspects of behaviour change may be beyond the individual’s control. This may include partner refusal—which is important for condom use, peer pressure, structural barriers and addictive effects of smoking. In Ghana, a documentary analysis of 24 curricula used for HIV/AIDs prevention education found that school-based informational materials were of poor quality, including factual errors and omitted information, possibly hindering behavioural change [39]. There is room to improve the accuracy of health information provided to female high school students and others who may influence their behaviours (e.g., partners, peers, parents, educators).

We found that students who perceived cervical cancer and HPV infection as serious (such as affecting their relationships and causing death) were less likely to engage in behaviours leading to cervical cancer and HPV infection high-risk classes. Consistent with Denny-Smith et al. [17] female college students who engaged in high-risk sexual behaviour had low perceived seriousness cervical cancer and HPV infection. Ensuring female senior high school students understand the seriousness of cervical cancer may be a useful target for health education, given that the knowledge-behaviour gap observed in other perceptual domains does not seem to extend to perceived severity. This may include providing education about the prevalence and incidence of cervical cancer, individualized estimates of risk, and consequences of the disease (e.g., medical, financial, and social consequences).

A range of potentially protective factors against engagement in risky behaviours leading to cervical cancer and HPV infection high-risk classes were identified in this study. First, students who are single (i.e., not dating) were less likely to belong to both high-risk cervical cancer and HPV infection classes. This finding could be explained by the low influence and reduced pressure to engage in risky behaviours as female high school students who are in relationships are more likely to be involved in sexual activities and contraceptive use [40].

Second, our study showed that living in a boarding house may reduce students’ likelihood of risky behaviours leading to cervical cancer; and this is consistent with findings from Kenya [41]. Contrary to this, in Brazil, boarding students were more likely to be involved in health risk behaviours, including sexual activities and smoking [42]. The differences in findings could be attributed to the level of freedoms and supervision. In Ghana, boarding schools have stringent policies providing fewer freedoms and adequate supervision to deter students; consequently, less permissive sexual behaviours. However, greater freedoms may exist (i.e., living away from home – perceived feeling of independence from and lack of accountability toward their family) in the boarding environment than in other settings internationally [42–44].

Third, similar to the boarding context in Ghana, students living with a parent are likely to experience some supervision, control and protection; this may explain their reduced likelihood of engagement in behaviours leading to HPV infection. This finding supports research from Africa and Europe, which points to the advantage of living with a parent [38, 45]. The collective findings from the monitoring role of parents and boarding school system in Ghana suggests that more controlled environment is less conducive to engaging in risky behaviours. However, the observed differences between African and Western settings warrant further research to understand and identify strategies for future interventions.

Finally, maternal employment status was found to be associated with HPV infection risk behaviours and this association has not been identified before in previous research [46]. It is plausible that this finding is likely to be illustrating the association between social status and risky behaviours, and suggests that risk reduction strategies be targeted to socially disadvantaged students.

### Implications for practice

This study provides information about the high prevalence of and factors influencing risky behaviours among female adolescents and young women leading to the contraction HPV infection and cervical cancer. There is a call for action to prioritise the implementation and strengthening of HPV vaccination and cervical cancer screening programmes by the government of Ghana to protect girls. The Ministry of Education and Ghana Education Service should expand the existing social studies or integrated science curriculum or proposed Comprehensive Sexuality and Reproductive Health Education to include cervical cancer and HPV infection.

While such curriculum changes may be implemented in the long term, and may require political will, increased advocacy by civil society organisations (such as non-governmental organisations, medical and nursing professional organisations) and general population grass root support, existing school activities could be leveraged in the short to medium term by heads of senior high schools. For example, planned and opportunistic school-based adolescent sexual health and education programmes including HIV and breast cancer by benevolent organisations, the Ghana AIDS commission and high school clubs should be expanded and strengthened to provide comprehensive behavioural cervical cancer and HPV infection risk reduction strategies to students. The existing Parent Teacher Association forums (consistently organised by every school) could be expanded to promote cervical cancer prevention programmes consistent with programmes directed at students within the school setting.

### Strengths and limitations

This is the first study to explore the cluster of risk factors for cervical cancer and HPV infection using a large sample size. However, there are some limitations. First, the results of this study were dependent on self-report, which is subject to recall bias. To reduce this bias, a reliable and validated tool was used to measure sexual behaviours, and findings were consistent with previous studies in Ghana.

Second, the prevalence of self-reported risk behaviours may be underestimated. This is because due to the sensitivity of the questions, cultural and religious expectations, social desirability bias is likely to occur. For instance, in Ghana, premarital sex is unaccepted, described as immoral and generates negative reactions. To minimise this bias, the importance of the study and participants’ confidentiality was emphasized during the information and anonymous survey sessions.

Finally, the findings of this study were restricted to public senior high school students aged 16 years and above within schools in the Ashanti region, reducing the generalisability of the results across other school settings in Ghana. While this is important, more than 65% of senior high schools in Ghana are government managed and admit the highest number of students with the Ashanti region recording the highest number of senior schools [23].

## Conclusions

There is a high prevalence and co-occurrence of cervical cancer and HPV infection risk factors with high and low risk groups identified among female senior high school students in Ghana. This study demonstrates that subjective perceptions of susceptibility and seriousness of cervical cancer and HPV infections may influence students’ engagement in risky behaviours; therefore, there is a need to support innovative integrated behavioural programmes aiming to increase risk perceptions. At the same time, the higher prevalence of risk factors among those perceiving a risk underlines the considerable barriers to engaging in cervical cancer prevention behaviours. Protective factors against exposure to cervical cancer and HPV infection risk factors are more controlled environment (i.e., school and parental supervision), maternal employment and being single. While comprehensive school-based multi-component risk reduction programmes for students are needed, complex interventions should target students in high-risk groups. Future research should include other regions of Ghana to enhance the generalisability and confirm the findings of the study. If similar patterns are observed, risk reduction interventions targeting specific clusters of risk factors should be developed and tested for female high school students.

## Supplementary Information

**Additional file 1.**

## Data Availability

The datasets used and/or analysed during the current study are available from the corresponding author upon reasonable request.
