# Supplementary material for: Prevalence and correlates of modifiable risk factors for cervical cancer and HPV infection among senior high school students in Ghana: a latent class analysis
Source: BMC Public Health. 2023 Feb 15;23:340. doi: 10.1186/s12889-022-14908-w (PMC9930033; doi:10.1186/s12889-022-14908-w)
Supplement: Supplementary file 1 — Additional file 1. [file 12889_2022_14908_MOESM1_ESM.docx]

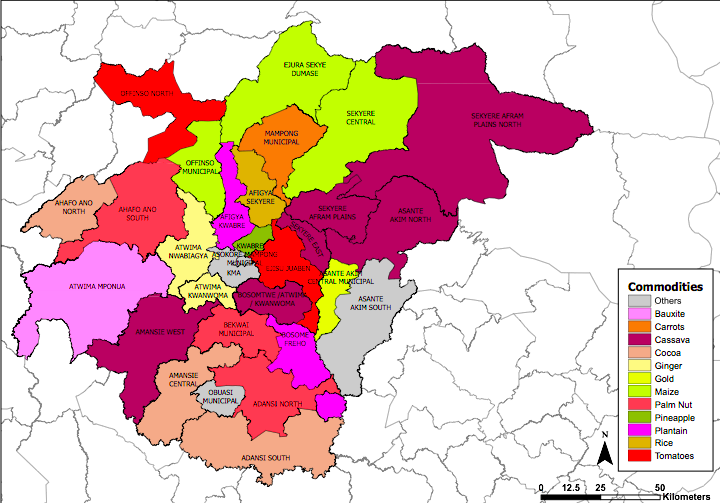


*Figure S1: Map of the Ashanti Region indicating division of Ashanti region into northern and southern sectors.* ***Photo credit:*** *Dwamena, H. A., Tawiah, K., & Akuoko Kodua, A. S. (2022). The Effect of Rainfall, Temperature, and Relative Humidity on the Yield of Cassava, Yam, and Maize in the Ashanti Region of Ghana.*

Supplementary file (S) 2: Cervical cancer survey

Participant ID: 🞎🞎🞎🞎

**SHiNE**

**The**

**project**

**Cervical Cancer Survey**

Version 3, dated 18032021

**SECTION A: Knowledge of cervical cancer**

*The following questions are about your knowledge of things or activities that can lead to cervical cancer. For each question, please tick the box (🗹) which best describes your answer. If you are unsure about a question, please give the best answer that you can.*

| 1. Which of the following can increase chances of getting cervical cancer*? Cervical cancer is a disease that can affect the tip of the womb.* | Yes | No |
| --- | --- | --- |
| 1. Smoking cigarettes | _1_🞎 | _2_🞎 |
| 1. Having sexually transmitted infections e.g., Human Papillomavirus, HIV/AIDS | _1_🞎 | _2_🞎 |
| 1. Using oral contraceptives | _1_🞎 | _2_🞎 |
| 1. Having one or more abortions | _1_🞎 | _2_🞎 |
| 1. Having sex before 18 years of age | _1_🞎 | _2_🞎 |
| 1. Applying Dettol or strong soaps into the vagina during bathing | _1_🞎 | _2_🞎 |
| 1. Having had more than one sexual partner over a lifetime | _1_🞎 | _2_🞎 |
| 1. Spiritual forces (e.g. witchcraft) | _1_🞎 | _2_🞎 |
| 1. Inserting herbs into the vagina | _1_🞎 | _2_🞎 |
| 1. Having a family history of cervical cancer | _1_🞎 | _2_🞎 |
| 1. Poor personal hygiene | _1_🞎 | _2_🞎 |
| 1. Contact with blood of a person with cervical cancer | _1_🞎 | _2_🞎 |
|  |  |  |
| 1. Which of the following can reduce a woman’s chance of getting cervical cancer? | **True** | **False** |
| 1. Praying to God | _1_🞎 | _2_🞎 |
| 1. Fasting to God | _1_🞎 | _2_🞎 |
| 1. Using a condom during sexual intercourse | _1_🞎 | _2_🞎 |
| 1. Having regular cervical check ups | _1_🞎 | _2_🞎 |
| 1. Getting vaccinated with HPV vaccines to prevent cervical cancer   (*A vaccine is a liquid often injected into the arm)* | _1_🞎 | _2_🞎 |

**SECTION B: Your thoughts and view about HPV and cervical cancer**

*The following questions explore your thoughts and views about HPV and cervical cancer. For each question, please tick the box (🗹) that describes your answer to each question. If you are unsure about a question, please give the best answer that you can. Remember that the answers you give will remain confidential.*

Please rate your chances of getting **HPV infection and cervical cancer.**

| About HPV | Very unlikely | Unlikely | Not sure | Likely | Very Likely |
| --- | --- | --- | --- | --- | --- |
| 1. How likely are you to get HPV infection in your life? | 1🞎 | 2🞎 | 3🞎 | 4🞎 | 5🞎 |
| 1. How likely is it that your sexual activities will lead you to getting HPV infection? | 1🞎 | 2🞎 | 3🞎 | 4🞎 | 5🞎 |
|  |  |  |  |  |  |
| About cervical cancer | **Very unlikely** | **Unlikely** | **Not sure** | **Likely** | **Very Likely** |
| 1. How likely are you to develop cervical cancer? | 1🞎 | 2🞎 | 3🞎 | 4🞎 | 5🞎 |
| 1. How likely is it that your family background will lead you to get cervical cancer? | 1🞎 | 2🞎 | 3🞎 | 4🞎 | 5🞎 |
| 1. How likely is it that your physical fitness will lead you to get cervical cancer? | 1🞎 | 2🞎 | 3🞎 | 4🞎 | 5🞎 |
| 1. How likely is it that your spirituality protects you from getting cervical cancer? | 1?🞎 | 2🞎 | 3🞎 | 4🞎 | 5🞎 |

Please rate your views about the impact of having **HPV infection and cervical cancer**

| Getting HPV would: | Strongly disagree | Disagree | Not sure | Agree | Strongly agree |
| --- | --- | --- | --- | --- | --- |
| 1. Make it difficult to get pregnant. | 1🞎 | 2🞎 | 3🞎 | 4🞎 | 5🞎 |
| 1. Interfere with my learning at school. | 1🞎 | 2🞎 | 3🞎 | 4🞎 | 5🞎 |
| 1. Affect my relationship with my boyfriend/girlfriend or husband | 1🞎 | 2🞎 | 3🞎 | 4🞎 | 5🞎 |
| 1. Result in serious health challenges. | 1🞎 | 2🞎 | 3🞎 | 4🞎 | 5🞎 |
|  |  |  |  |  |  |
| The thought of cervical cancer: | **Strongly disagree** | **Disagree** | **Not sure** | **Agree** | **Strongly agree** |
| 1. Makes me scared. | 1🞎 | 2🞎 | 3🞎 | 4🞎 | 5🞎 |
| 1. Makes me panic. | 1🞎 | 2🞎 | 3🞎 | 4🞎 | 5🞎 |
| 1. Makes me sad | 1🞎 | 2🞎 | 3🞎 | 4🞎 | 5🞎 |
| 1. Makes me worry | 1🞎 | 2🞎 | 3🞎 | 4🞎 | 5🞎 |
|  |  |  |  |  |  |
| Getting cervical cancer would: | **Strongly disagree** | **Disagree** | **Not sure** | **Agree** | **Strongly agree** |
| 1. Reduce the number years I live | 1🞎 | 2🞎 | 3🞎 | 4🞎 | 5🞎 |
| 1. Affect my relationships with the people close to me. | 1🞎 | 2🞎 | 3🞎 | 4🞎 | 5🞎 |
| 1. Make me die | 1🞎 | 2🞎 | 3🞎 | 4🞎 | 5🞎 |

**SECTION C: Sexual behavior**

*The following questions are about your sexual behavior. For each question, please tick the box (🗹) which best describes your answer. If you are unsure about a question, please give the best answer that you can. Remember that the answers you give will remain confidential.* ***Note: these questions are optional.***

| 1. Have you had sexual intercourse before? | _1_🞎 Yes  _2_🞎 No  _3_🞎 Prefer not to answer |
| --- | --- |
| 1. How old were you the first time you had sexual intercourse? | _1_🞎 I have never had sexual intercourse before  _2_🞎 16 years of age or younger  _3_🞎 17 years of age or older  _4_🞎 Prefer not to answer |
| 1. During your life, how many people have you had sexual intercourse with? | _1_🞎 I have never had sexual intercourse before  _2_🞎 one person  _3_🞎 two or more people  _4_🞎 Prefer not to answer |
| 1. The last time you had sexual intercourse, did you or your partner use a condom? | _1_🞎 I have never had sexual intercourse before  _2_🞎 Yes  _3_🞎 No  _4_🞎 Prefer not to answer |
| 1. During the past 12 months, have you been tested with a sexually transmitted infection e.g. gonorrhoea, syphilis, chlamydia, HIV e.t.c,? | _1_🞎 Yes  _2_🞎 No  _3_🞎Prefer not to answer |
| 1. The last time you had sexual intercourse, did you use birth control pills to prevent pregnancy? | _1_🞎 I have never had sexual intercourse before  _2_🞎 Yes  _3_🞎 No  _4_🞎 Prefer not to answer |

**SECTION D: Information about you**

*The following questions are about you. For each question, please tick the box (🗹) which best describes your answer or write your answer in the space provided. If you are unsure about a question, please give the best answer that you can. Remember that the answers you give will remain confidential*.

| 1. What is your age? | ………………years | |
| --- | --- | --- |
| 1. What is your ethnicity? | _1_🞎 Akan  _2_🞎 Northner  _3_🞎 Ewe/Guans  _4_🞎 Ga – Adangbe  _5_🞎Other (please specify)……………………… | |
| 1. What is your relationship status? | _1_🞎 Single  _2_🞎 Dating  _3_🞎 Other (please specify)……………………… | |
| 1. What program are you studying at school? | _1_🞎Science  _2_🞎General arts  _3_🞎Visual arts  _4_🞎Business  _5_🞎Home economics  _6_🞎Agric | |
| 1. What is your enrolment status at school? | _1_🞎 Boarding student  _2_🞎 Day student | |
| 1. Who do you live with most of the time during school holidays? | _1_🞎 Both parents  _2_🞎 One parent  _3_🞎 Grandparent (s)  _4_🞎 Guardian  _5_🞎 Friend(s)  _6_🞎 On my own | |
| 1. What is the occupation of your father or male guardian? | _1_🞎 Unemployed  _2_🞎Employed…………………..please specify | |
| 1. What is the occupation of your mother or female guardian? | _1_🞎 Unemployed  _2_🞎 Employed…………………. please specify | |
| 1. Have you smoked cigarettes, marijuana “weed?”, or shisha in the before? | _1_🞎 Yes  _2_🞎 No |  |

**Thank you for completing this survey. Your time is greatly appreciated.**

**If you have any comments, please write them in the space below.**

………………………………………………………………………………………………………………………………………………………………………………………………………………………………………………………………………………………………………………………………………………………………………………………………………………………………………………………………
